# An Accurate Tool for Uncovering Cancer Subtypes by Fast Kernel Learning Method to Integrate Multiple Profile Data

**DOI:** 10.3389/fcell.2021.615747

**Published:** 2021-03-05

**Authors:** Hongyu Zhang, Limin Jiang, Jijun Tang, Yijie Ding

**Affiliations:** ^1^School of Computer Science and Technology, College of Intelligence and Computing, Tianjin University, Tianjin, China; ^2^Department of Computer Science and Engineering, University of South Carolina, Columbia, SC, United States; ^3^School of Electronic and Information Engineering, Suzhou University of Science and Technology, Suzhou, China

**Keywords:** cancer subtypes classification, SVM, multiple kernel learning, gene expression profile, isoform expression, DNA methylation

## Abstract

In recent years, cancer has become a severe threat to human health. If we can accurately identify the subtypes of cancer, it will be of great significance to the research of anti-cancer drugs, the development of personalized treatment methods, and finally conquer cancer. In this paper, we obtain three feature representation datasets (gene expression profile, isoform expression and DNA methylation data) on lung cancer and renal cancer from the Broad GDAC, which collects the standardized data extracted from The Cancer Genome Atlas (TCGA). Since the feature dimension is too large, Principal Component Analysis (PCA) is used to reduce the feature vector, thus eliminating the redundant features and speeding up the operation speed of the classification model. By multiple kernel learning (MKL), we use Kernel target alignment (KTA), fast kernel learning (FKL), Hilbert-Schmidt Independence Criterion (HSIC), Mean to calculate the weight of kernel fusion. Finally, we put the combined kernel function into the support vector machine (SVM) and get excellent results. Among them, in the classification of renal cell carcinoma subtypes, the maximum accuracy can reach 0.978 by using the method of MKL (HSIC calculation weight), while in the classification of lung cancer subtypes, the accuracy can even reach 0.990 with the same method (FKL calculation weight).

## Introduction

Cancer is one of the most severe diseases endangering human life and health in the world. Among them, lung cancer and kidney cancer, which are the top ten killers of cancer, are the leading causes of cancer death. Lung cancer includes small cell lung cancer (SCLC) and non-small cell lung cancer (NSCLC). Lung adenocarcinoma (LUAD) and Lung squamous cell carcinoma (LUSC), two subtypes of NSCLC, accounting for about 85% of lung cancer (Herbst et al., 2018). Among the common types of renal cell carcinoma (RCC), Kidney renal clear cell carcinoma (KIRC) (75–80%), Kidney renal papillary cell carcinoma (KIRP) (10–15%), and Kidney Chromophobe (KICH) (5%) account for the vast majority. Correct diagnosis of cancer subtypes is helpful to find potential therapeutic targets and new drug development, so that reduce the mortality of cancer. At present, it is challenging to classify subtypes by traditional pathological analysis. The mature sequencing technology makes it possible for people to obtain a large number of gene expression profiles. According to the above gene expression data, early diagnosis of cancer can be made based on gene expression profiles even if some tissues of organisms have not changed significantly. Moreover, it also provides an excellent help for the classification of cancer subtypes.

In the classification of NSCLC subtypes, some studies have shown that the characteristics of mRNA expression or gene histology contribute to the conventional histopathological classification (Jun, 2010; Girard et al., 2016). In addition, we can also consider the relationship between genes, not just individual genes (Su et al., 2019). However, in the subtype classification of renal cell carcinoma, MiRNA signature obtained using quantitative reverse transcription-polymerase chain reaction (QRT-PCR) analysis has been proved to be effective in the classification of RCC subtypes (Youssef et al., 2011). Furthermore, using ensemble classification methods can get better results than a single machine learning algorithm (Park et al., 2018). All methods only focus on one feature of cancer or the correlation of one feature to classify cancer subtypes, while ignoring the influence of other characteristics.

At present, computational methods (Zeng et al., 2018; Qi et al., 2019) have been widely applied to biological problems. It is mainly divided into two directions: one is the traditional machine learning method, which includes mostly Logistic Regression (LR), K-Nearest Neighbor (KNN), RF (Random Forest), SVM, etc.; and the other is multiple kernel learning (Ding et al., 2020b; Guo et al., 2020; Liu et al., 2020; Zou et al., 2020). It maps different feature components of heterogeneous data with different kernel functions so that the data can be better expressed in the new feature space, and the classification performance is significantly improved. Multiple kernel learning has been widely used in the field of computational biology, for example, protein function identification (Ding et al., 2020a), drug-side effect association (Ding et al., 2018; Yijie et al., 2019), drug-target interactions (Ding et al., 2019), etc.

**FIGURE 1:**
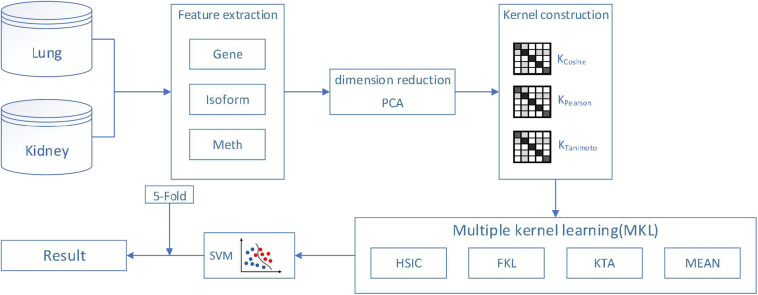
The flowchart of our proposed method.

In this paper, we use the classical machine learning algorithm SVM to classify cancer subtypes based on gene expression, isoform expression and methylated expression. Because gene expression profiles are generally obtained by gene chip sequencing in biological systems. However, there are a huge number of genes in the cells of organisms, so the microarray data we get will also present the characteristics of small samples, high latitude, high noise, uneven distribution and so on. Therefore, it is necessary to use the PCA method to extract effective data from massive cancer features. It is worth mentioning that PCA is used to reduce the dimension of features data used in this experiment, and the dimension of features can differ by thousands of times. In addition, we use cross-validation method to make the algorithm more robust and get more accurate and reliable results. Finally, we construct the kernel function by features, and then apply multiple kernel learning (weight is calculated by HSIC, FKL, KTA, mean methods) to combine the kernel functions of multiple features into SVM, to get more excellent results. The flowchart is shown in Figure 1.

In the classification of lung cancer and kidney cancer subtypes, we have achieved the excellent results (the accuracy of kidney cancer: 0.978, the accuracy of lung cancer: 0.990). Identifying more accurate cancer subtypes will not only help to provide more appropriate individualized treatment suggestions for diseases in real life, but also promote the discovery of potential therapeutic targets and the development of new drugs, so as to improve the survival rate of patients.

In the section of “Materials and Methods,” we mainly introduced the data source, subtype types and data set size of lung cancer and renal cancer. Next, the dimension reduction method PCA and machine learning algorithm SVM used in the experiment are described in detail. Then, it is introduced the feature kernel construction method applied in SVM and the kernel fusion method applied in multi-kernel learning. The next section result shows the method of establishing model and evaluation criteria used in the experiment. After that, we draw the TSNE visualization graph of the reduced features and show the experimental results of different features in random forest, different classifiers, single-kernel SVM and multi-kernel SVM. In the last section of the paper, the conclusion summarizes the specific process of the experiment and the prospect of the paper.

## Materials and Methods

### Datasets

We obtain the cancer subtype data from the experiment in Broad GDAC (Xiao and Yang, 2016), which collects and analyzes the standardized data extracted from The Cancer Genome Atlas (TCGA) (Tomczak et al., 2015). We extract two cancer datasets, including renal cancer (RCC) and non-small cell lung cancer (NSCLC). RCC has three subtypes: Kidney Chromophobe (KICH), Kidney renal clear cell carcinoma (KIRC), Kidney renal papillary cell carcinoma (KIRP), where 113 samples in KICH, 537 in KIRC and 323 in KIRP. NSCLC has two subtypes: Lung adenocarcinoma (LUAD) and Lung squamous cell carcinoma (LUSC), where 585 samples in LUAD and 504 in LUSC. In addition, we remove the redundant data from the features (retain the cancer samples with sample number of 01–09), and get the sample cases with three dimensions information (gene expression, isoform expression and methylated expression) at the same time. We added tag information to each case. Finally, the lung cancer data set consists of two background subtypes, LUSC and LUAD, with a total sample number of 824; the renal cancer data set consists of KICH, KIRC and KIRP, with a total number of 658 samples.

### Feature Dimension Reduction

**FIGURE 2:**
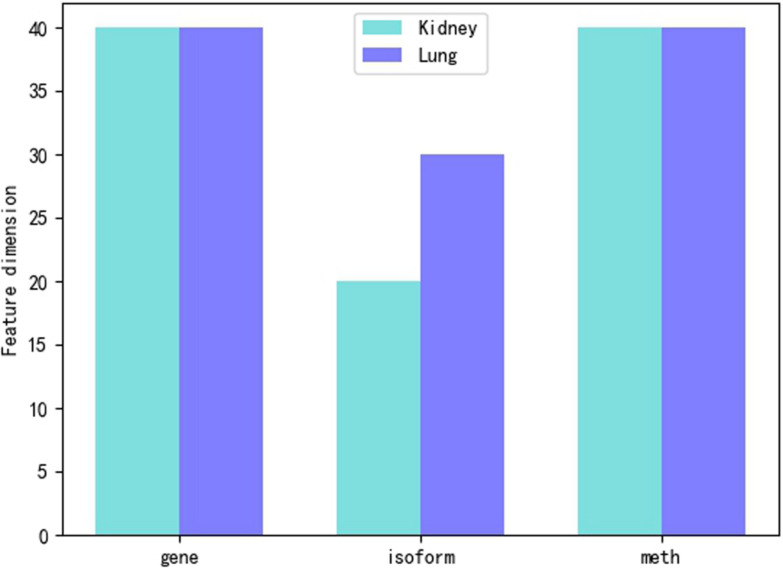
Feature dimension reduction.

We use three different feature sets, such as gene expression, isoform expression and methylation expression. However, the feature dimensions are too large that the smallest dimension has reached more than 20,000, so it is necessary to reduce the dimension of features. We mainly use Principal Component Analysis (PCA) (Jolliffe, 2002) to reduce characteristic dimensions of gene expression, isoform expression and methylated expression. We find that the dimension of gene and meth feature for kidney and lung is the best at about 40. Compared with isoform feature, the dimension of kidney feature is reduced to 20, and the dimension of lung feature is reduced to 30. The dimension of feature reduction can be shown in Figure 2.

### Support Vector Machine

Support Vector Machine (SVM) was proposed by Cortes and Vapnik (1995), by calculating the maximum margin hyperplane, be mainly applied to classification and regression problems. SVM is one of the kernel learning methods. It can be used to solve non-linear problems by mapping the low-dimensional feature to high-dimensional space.

In our experiment, we mainly use Pearson Correlation Coefficient, Cosine Similarity and Tanimoto Similarity to make kernel function, and build multi-kernel learning method to calculate the weights of kernel function through HSIC, KTA, FKL, and Mean models, so as to classify the subtype data of renal cancer and lung cancer.

### Kernel Construction

#### Tanimoto Similarity

Tanimoto similarity (Rogers and Tanimoto, 1960) is mainly used to calculate the similarity between individuals measured by symbols or boolean values. The larger the coefficient value is, the higher the sample similarity is. Now it has been widely used in biological data analysis. If the value is a binary vector, then the Tanimoto similarity coefficient is equal to the Jaccard distance. The tanimoto similarity can be computed as Eq. 1:

(1) $$\begin{array}{c} E_{\text{j}}\,\left(A,B\right)=\frac{A\cdot B}{{\left|A\right|}^{2}+{\left|B\right|}^{2}-A\cdot B}\\ A\cdot B=\sum_{i}A_{i}\,B_{i}\,{\left|A\right|}^{2}=\sum_{i}A_{\text{i}}^{2} \end{array}$$

where A and B are two vectors, *A*⋅*B* representing vector product, |*A*|^2^ and |*B*|^2^ are norms of the vector.

#### Pearson Correlation Coefficient

Pearson correlation coefficient (Williams, 1996) reflects the degree of correlation between the two variables, with the value between [−1, 1]. As the correlation coefficient approaches the value of -1 or 1, the linear relationship increases. If the coefficient is equal to 0, there is no linear relationship. Pearson correlation coefficient is expressed in the mathematical formula (2) as the covariance of two variables divided by the standard deviation of two variables as follows:

(2) $$\rho_{a,b}=\frac{c\,o\,v\,\left(a,b\right)}{\sigma_{a}\,\sigma_{b}}$$

where cov (a,b) is the covariance of variables a and b, σ_a_σ_b_ represents the standard deviation of variables a and b, respectively.

#### Cosine Similarity

Cosine similarity is used to measure the difference between two individuals. Compared with the first two kernel function making methods, cosine similarity pays more attention to the difference between vector positions in direction than in distance or length. In essence, it is to calculate the cosine value of the angle between two vectors, which can be expressed as Eq. 3:

(3) $$\text{s}\,\left(M,N\right)=\cos\theta =\frac{\vec{m}\cdot \vec{n}}{\left|m\right|\cdot \left|n\right|}$$

### Multiple Kernel Learning

Compared with a single kernel function, multi-kernel learning (Gönen and Alpaydın, 2011) must be more flexible and developable. The mapping space of multiple kernel function is composed of the feature space for each single kernel function. Obviously, the combination space can map the different feature components of heterogeneous data through a suitable kernel fusion model, which has more accurate and reasonable expression, so as to improve the classification accuracy. We mainly use the multi-kernel linear combination method, which is essentially a linear combination of all kernel functions. Given_*K_i_*_ is the i-th kernel matrix,_β_i__ is the i-th weight of the matrix, the multi-kernel fusion function can be described as Eq. 4:

(4) $$K=\sum_{\text{i}}\beta_{\text{i}}\,K_{i}$$

$$\sum_{\text{i}}\beta_{i}=1\,\beta_{\text{i}}\geq 0$$

#### Hilbert-Schmidt Independence Criterion

Hilbert-Schmidt Independence Criterion (HSIC) is to measure the distribution difference between two variables (Gretton et al., 2005; Wang et al., 2016), which is similar to covariance. The construction method depends on the covariance operator in Hilbert space as Eq. 5:

(5) $$H\,S\,I\,C\,\left(Z^{\left(a\right)}\,Z^{\left(b\right)}\right)={\left(n-1\right)}^{-2}\,t\,r\,\left(K_{a}\,H\,K_{b}\,H\right)$$

where *Z*^(*a*)^ and *Z*^(b)^ are two different data sets, n is the number of samples, tr(x) is the trace of matrix X,_*K_a_*,*K_b_*,*H∈ℜ^m×m^*,*K_a_*,*K_b_*_ is gram matrix of data set, $H_{i\,j=}\,\delta_{i\,j}-\frac{1}{m}$ is a matrix with a mean value of 0.

#### Fast Kernel Learning

Referring to the description of fast kernel learning (FKL) (Shen et al., 2019a), in multi-kernel learning, we think that the target similarity matrix K should be close to the label similarity matrix Y, where_*Y=yy^T^*_. To prevent overfitting, we usually add a regularization term_|α|^2^_. Therefore, the solution of multi-kernel fusion weights can be sorted into a quadratic programming problem as Eq. 6:

(6) $$\underset{\alpha ,\text{k}}{\text{min}}{\left|K-Y\right|}_{F}^{2}+\lambda \,{\left|\alpha \right|}^{2}$$

$$s.t.\;\sum_{\text{w}=1}^{J}\alpha_{\text{w}}=1$$

Where F is Frobenius norm, λ is an equilibrium coefficient, and J is the number of kernel functions.

The formula can be further derived. Since the Frobenius norm of a matrix is equal to the trace of the product of the matrix and its transposed matrix, $\left||{\text{A||}}_{\text{F}}^{2}=\text{tr}({\text{AA}}^{\text{T}}\right)$. The formula can be simplified as Eq. 7:

$$\underset{\alpha }{\text{min}}\alpha^{T}\,\left(A+\lambda \,I\right)\,\alpha -2\,b^{T}\,\alpha$$

(7) $$s.t.\sum_{\text{w}}^{J}\alpha_{\text{w}}=1$$

$$\alpha_{\text{w}}\geq 0,w=1,\ldots ,J$$

Here I is the identity matrix of the same size as A.

#### Kernel Target Alignment

Kernel target alignment (KTA) (Shen et al., 2019b) is mainly used to calculate the weight of kernel in multi-core learning. If_wα_ is used to represent the score of KTA, the greater the value of_w_α__, the greater the correlation between the two kernels, which makes a higher contribution to the composite kernel with_*F_train_*_ aligned kernel matrix, and vice versa. The alignment between *K*_α_ and_*K_ideal_*_ is called kernel target alignment (KTA). The ideal kernel matrix (_*K_ideal_*_) is calculated as Eq. 8:

(8) $$K_{\text{ideal}}=F_{\text{train}}\,F_{\text{train}}^{T}$$

Fraction formula (9) for calculating KTA:

(9) $${\text{w}}_{\alpha }=\frac{{\left\langle K_{\alpha },K_{\text{ideal}}\right\rangle }_{F}}{{\left|K_{\alpha }\right|}_{F}\,{\left|K_{\text{ideal}}\right|}_{F}}$$

Where 〈X,Y〉*_F_* represents Frobenius inner product Trace (⋅)._|X|_F__ stands for Frobenius norm.

## Results

### Cross Validation

Cross validation can effectively avoid overfitting and improve the generalization ability of the model. Main idea is to divide the dataset into N subsets, randomly select N-1 subsets as the training set, the rest as the prediction set, to get the performance evaluation index of the classifier. This process continues until all subsets are predicted and only once. The final model evaluation results are obtained by combining N evaluation results. In our experiment, we mainly use the twofold cross validation to train and evaluate our model.

### Evaluation Metrics

We evaluate the classifier based on sensitivity (SN), specificity (SP), accuracy (ACC), Mathew’s correlation coefficient (MCC) as Eqs 10a–d.

(10a) $$S\,N=\frac{T\,P}{T\,P+T\,N}$$

(10b) $$S\,P=\frac{T\,N}{T\,N+F\,P}$$

(10c) $$A\,C\,C=\frac{T\,P+T\,N}{T\,P+T\,N+F\,P+F\,N}$$

(10d) $$M\,C\,C=\frac{T\,P\times T\,N-F\,P\times F\,N}{\sqrt{\left(T\,P+F\,P\right)\times \left(T\,P+F\,N\right)\times \left(T\,N+F\,P\right)\times \left(T\,N+F\,N\right)}}$$

where TP, FP denote true positive, false positive; TN, FN denote true negative, false negative.

In addition, we draw the receiver operating characteristic (ROC) curves to better describe the data. We also get the area under curve (AUC) values by calculating the area of the ROC curve.

### Analysis of Feature Dimension Reduction

Three feature sets, such as gene expression profile (gene), isoform expression (isoform), DNA methylation data (meth), construct kernel functions by cosine similarity (cosine), pearson correlation-based similarity (pearson), tanimoto similarity coefficient (tanimoto), and then cross-validation in SVM. It is necessary to reduce the dimension of the feature on the premise of slightly losing accuracy.

#### Tsne Feature Visualization

**FIGURE 3:**
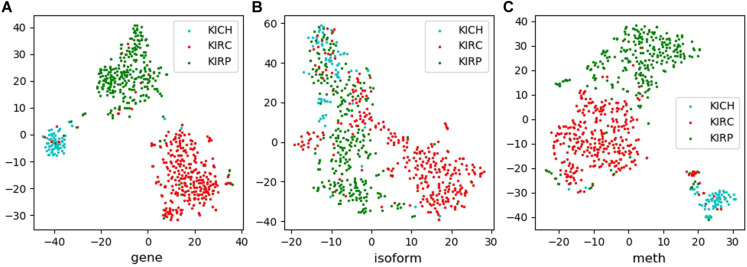
Feature visualizations on RCC. **(A)**: feature set of gene expression profile; **(B)**: feature set of isoform expression; **(C)**: feature set of DNA methylation.

**FIGURE 4:**
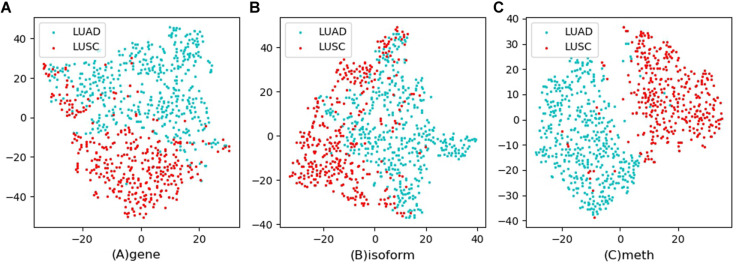
Feature visualizations on NSCLC. **(A)**: feature set of gene expression profile; **(B)**: feature set of isoform expression; **(C)**: feature set of DNA methylation.

Tsne is a non-linear dimensionality reduction method, which can map the high-dimensional feature data to the low-dimensional space, so that it can be visualized in the graph. Feature visualizations on RCC and NSCLC are shown in Figures 3, 4.

#### Performance of Various Classifiers

**FIGURE 5:**
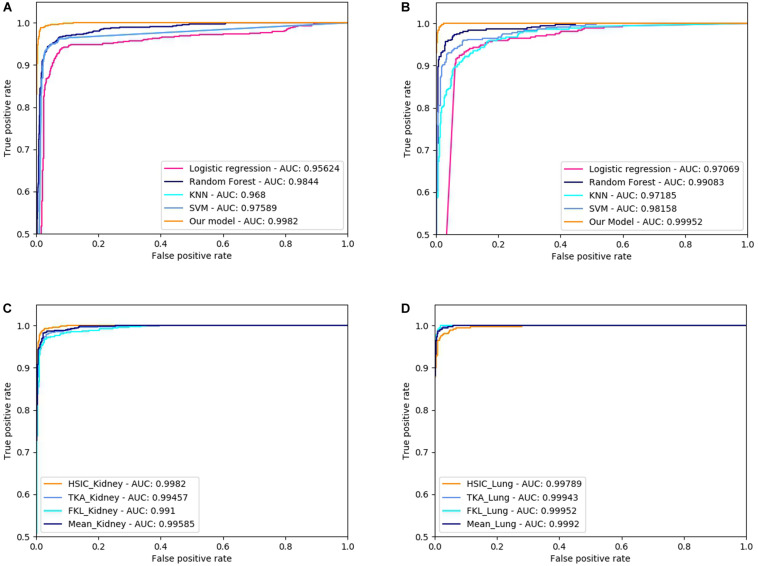
ROC curve of different classifiers and difference methods of kernel fusion on RCC and NSCLC. **(A)** The ROCs of different classifiers on Kidney cancer dataset; **(B)** The ROCs of different classifiers on Lung cancer dataset; **(C)** The ROCs of different MKL algorithms on Kidney cancer dataset; **(D)** The ROCs of different MKL algorithms on Lung cancer dataset.

Three dimensionally reduced features are put into random forest to get the results of Table 1. The best result of the kidney is gene feature with ACC of 0.951 and MCC of 0.917, and the best result of lung is meth feature with ACC of 0.973 and MCC of 0.946. All reduction features are feed into four classifiers, such as SVM, RF, KNN, LR, as shown in Table 2. Moreover, we also plot ROC curve to better describe the data, as shown in Figures 5A,B.

**TABLE 1:** Results of different characteristics in random forest after dimensionality reduction.

RF		SN	SP	ACC	MCC	AUC
Kidney	Gene	0.951	0.975	0.951	0.917	0.986
	Isoform	0.927	0.963	0.927	0.875	0.979
	Meth	0.946	0.973	0.946	0.909	0.992
Lung	Gene	0.971	0.883	0.932	0.864	0.976
	Isoform	0.951	0.862	0.911	0.821	0.964
	Meth	0.977	0.967	0.973	0.946	0.996

**TABLE 2:** Results of different classifiers after feature dimension reduction.

SVM	SN	SP	ACC	MCC	AUC
Kidney	0.940	0.970	0.940	0.899	0.975
Lung	0.980	0.9	0.944	0.888	0.981

**RF**	**SN**	**SP**	**ACC**	**MCC**	**AUC**

Kidney	0.924	0.962	0.924	0.871	0.984
Lung	0.982	0.929	0.958	0.917	0.990

**KNN**	**SN**	**SP**	**ACC**	**MCC**	**AUC**

Kidney	0.936	0.968	0.936	0.893	0.968
Lung	0.951	0.867	0.913	0.826	0.971

**LR**	**SN**	**SP**	**ACC**	**MCC**	**AUC**

Kidney	0.898	0.949	0.898	0.831	0.956
Lung	0.929	0.918	0.924	0.849	0.970

### Evaluation of Kernel Construction

After dimensionality reduction, three feature sets are constructed by different kernel construction methods as cosine, pearson and tanimoto, and put into SVM classifier for cross- validation. Results are shown in Table 3. It is not difficult to find that the dimensionality reduction features can be used to classify cancer subtypes.

**TABLE 3:** Results of different kernel functions with different features in SVM.

Kidney		SN	SP	ACC	MCC	AUC
Gene	Cosine	0.955	0.978	0.955	0.925	0.984
	Pearson	0.955	0.978	0.955	0.925	0.984
	Tanimoto	0.957	0.978	0.957	0.927	0.988
Isoform	Cosine	0.948	0.974	0.948	0.913	0.979
	Pearson	0.948	0.974	0.948	0.913	0.981
	Tanimoto	0.936	0.968	0.936	0.892	0.973
Meth	Cosine	0.980	0.990	0.980	0.966	0.997
	Pearson	0.978	0.989	0.978	0.964	0.998
	Tanimoto	0.975	0.987	0.975	0.958	0.997

**Lung**		**SN**	**SP**	**ACC**	**MCC**	**AUC**

Gene	Cosine	0.986	0.908	0.951	0.903	0.983
	Pearson	0.982	0.905	0.947	0.895	0.984
	Tanimoto	0.971	0.924	0.950	0.900	0.982
Isoform	Cosine	0.975	0.913	0.947	0.895	0.983
	Pearson	0.971	0.918	0.947	0.895	0.983
	Tanimoto	0.969	0.916	0.945	0.890	0.983
Meth	Cosine	0.989	0.994	0.991	0.982	0.998
	Pearson	0.988	0.986	0.987	0.975	0.999
	Tanimoto	0.993	0.994	0.993	0.987	0.999

### Evaluation of Multiple Kernel Fusion

The characteristic gene + tanimoto, isoform + pearson, meth + cosine kernels of Kidney with the best results are fused by HSIC, KTA, FKL, and Mean weighted methods, and it is found that HSIC fusion method has better effect. The characteristic gene + cosine, isoform + cosine and meth + tanimoto of Lung with the best results are fused by HSIC, KTA, FKL, and Mean weighted methods, and it is found that FKL fusion method is the best. All results are shown in Table 4. Also, we plot ROC curves for four different methods of kernel fusion shown in Figures 5C,D.

**TABLE 4:** Results of different kernel fusion methods in SVM.

Kidney	SN	SP	ACC	MCC	AUC
HSIC	0.978	0.989	0.978	0.964	0.998
FKL	0.957	0.978	0.957	0.927	0.990
KTA	0.963	0.981	0.963	0.938	0.994
Mean	0.966	0.983	0.966	0.943	0.995

**Lung**	**SN**	**SP**	**ACC**	**MCC**	**AUC**

HSIC	0.978	0.972	0.975	0.951	0.997
FKL	0.988	0.991	0.990	0.980	0.999
KTA	0.988	0.989	0.989	0.978	0.999
Mean	0.988	0.986	0.987	0.975	0.999

## Conclusion

In this paper, we obtained the data of two cancer subtypes (lung cancer and renal cancer) from Broad GDAC Firehouse, which collections and analyses the standardized data extracted from TCGA. We use Principal Component Analysis (PCA) method to reduce the dimension of features. The features are constructed into kernel functions by using cosine, pearson, tanimoto and other similarity measurement methods. Then the multiple kernel learning (MKL) method (KTA, FKL, HSIC, mean to calculate the weight of kernel fusion) is used to combine multiple kernel functions into a combined kernel function. Finally, the calculated kernel function is put into SVM to predict cancer subtypes. In addition, compared our model with some commonly used machine learning algorithms, such as random forest, linear regression, LR, and so on, our model has achieved good results. Our method also has some limitations. For example, we also calculated the feature data before dimension reduction by the above method. The results show that the characteristic dimension of the data after dimension reduction is reduced by several thousand times compared with that before dimension reduction. Still, the accuracy is also reduced by 3–4 percentage points. How to reduce the feature dimension while minimizing the gap with the classification result before dimension reduction is a problem we will consider in the future.

## Data Availability Statement

The original contributions presented in the study are included in the article/supplementary material, further inquiries can be directed to the corresponding author/s.

## Author Contributions

YD, LJ, and HZ conceived and designed the experiments. HZ and LJ performed the experiments and analyzed the data. HZ and YD wrote the manuscript. YD and JT supervised the experiments and reviewed the manuscript. All authors have participated in study discussion and manuscript preparation and read and approved the final manuscript.

## Conflict of Interest

The authors declare that the research was conducted in the absence of any commercial or financial relationships that could be construed as a potential conflict of interest.
